# Sulforaphane regulates cell proliferation and induces apoptotic cell death mediated by ROS-cell cycle arrest in pancreatic cancer cells

**DOI:** 10.3389/fonc.2024.1442737

**Published:** 2024-08-29

**Authors:** Yongmin Cho, Moon Nyeo Park, Min Choi, Tarun Kumar Upadhyay, Han Na Kang, Jeong Min Oh, Soonki Min, Ji-Ung Yang, Moonkyoo Kong, Seong-Gyu Ko, Md Ataur Rahman, Abdel Halim Harrath, Bonglee Kim

**Affiliations:** ^1^ Department of Pathology, College of Korean Medicine, Kyung Hee University, Seoul, Republic of Korea; ^2^ Korean Medicine-Based Drug Repositioning Cancer Research Center, College of Korean Medicine, Kyung Hee University, Seoul, Republic of Korea; ^3^ Department of Biotechnology, Parul Institute of Applied Sciences and Research and Development Cell, Parul University, Vadodara, Gujarat, India; ^4^ KM Convergence Research Division, Korea Institute of Oriental Medicine, Daejeon, Republic of Korea; ^5^ Division of Lung and Head and Neck Oncology, Department of Radiation Oncology, Kyung Hee University Medical Center, Kyung Hee University College of Medicine, Seoul, Republic of Korea; ^6^ Department of Neurology, University of Michigan, Ann Arbor, MI, United States; ^7^ Department of Zoology, College of Science, King Saud University, Riyadh, Saudi Arabia

**Keywords:** pancreatic cancer, sulforaphane (SFN), reactive oxygen species (ROS), mitochondrial membrane potential (ΔΨm), sub G1, γH2A.X, apoptosis

## Abstract

**Background:**

Pancreatic cancer (PC), sometimes referred to as pancreatic ductal adenocarcinoma (PDAC), is a major cause of global mortality from cancer. Pancreatic cancer is a very aggressive and devastating kind of cancer, characterized by limited options for therapy and low possibilities of survival. Sulforaphane (SFN), a naturally occurring sulfur-containing compound, is believed to possess anti-inflammatory, anti-obesity, and anti-cancer characteristics.

**Objective:**

However, efficient preventative and treatment measures are essential and SFN has been studied for its ability to suppress pancreatic cancer cell proliferation and induce apoptosis.

**Methods:**

Here, SFN induced cytotoxicity and apoptosis in PDAC cell lines such as MIA PaCa-2 and PANC-1 cells, as evaluated by cytotoxicity, colony formation, western blot analysis, fluorescence-activated cell sorting (FACS), reactive oxygen species (ROS) detection, caspase-3 activity assay, immunofluorescence assay, and mitochondrial membrane potential assay.

**Results:**

In MIA PaCa-2 and PANC-1 cells, SFN inhibited cell survival and proliferation in a dose-dependent manner. The activation of caspase zymogens results in cleaved PARP and cleaved caspase-3, which is associated with an accumulation in the sub G1 phase. Furthermore, SFN increased ROS level and γH2A.X expression while decreasing mitochondrial membrane potential (ΔΨm). Notably, the ROS scavenger N-Acetyl-L-cysteine (NAC) was shown to reverse SFN-induced cytotoxicity and ROS level. Subsequently, SFN-induced cell cycle arrest and apoptosis induction as a Trojan horse to eliminate pancreatic cancer cells via ROS-mediated pathways were used to inhibit pancreatic cancer cells.

**Conclusion:**

Collectively, our data demonstrates that SFN-induced cell death follows the apoptosis pathway, making it a viable target for therapeutic interventions against pancreatic cancer.

## 1 Introduction

Pancreatic cancer (PC) is considered one of the most challenging opponents in the field of current cancer treatment (1). PC is characterized by the uncontrolled multiplication of cells in the pancreas, resulting in the formation of a tumor (2, 3). The elusive and hard-to-detect characteristics of this condition make it a formidable obstacle for both patients and healthcare professionals, frequently remaining undiagnosed until it reaches an advanced stage. The pancreas is an endocrine and exocrine gland situated posterior to the stomach, responsible for the secretion of hormones and digesting enzymes (4). Traditional treatments, although providing temporary relief, typically fail to effectively address its unrelenting progression. PC can arise from two distinct cell types: exocrine as well as neuroendocrine (5). Exocrine cells are more prevalent and frequently encountered in an advanced stage (6). However, early detection of pancreatic cancer poses a challenge due to its elusive nature, and symptoms typically manifest only in the later stages of the disease (7, 8). Therefore, researchers continue to hunt for new treatment approaches, motivating them to investigate the unexplored capabilities of natural substances such as sulforaphane.

Sulforaphane (SFN), a sulfur-rich chemical found in significant quantities in cruciferous vegetables like broccoli, Brussels sprouts, along with kale, has been highly esteemed for its beneficial effects on health (9, 10). Recent research has discovered that sulforaphane has the capacity to trigger a series of complex chemical processes in pancreatic cancer cells, ultimately leading to their destruction through pathways involving reactive oxygen species (ROS) (11). Furthermore, SFN’s impact goes beyond simple growth inhibition (12). It has an extraordinary capacity to trigger apoptosis, the programmed cell death that is crucial for maintaining tissue homeostasis (12). In pancreatic cancer, the disruption of apoptotic pathways gives malignant cells a better chance of survival, allowing them to grow without obstacles (13). In this context, sulforaphane is identified as a precursor of cell death, initiating a series of molecular events that ultimately lead to the destruction of cancer cells, so restoring the intricate equilibrium between life and death (14). Sulforaphane’s anticancer effects are mostly attributed to its ability to regulate the complex process of the cell cycle (15). Disruptions in cell cycle progression in pancreatic cancer led to unregulated proliferation, which drives tumor growth (16). However, SFN precisely interferes with the cell cycle, effectively halting it at crucial stages in a coordinated manner (12). SFN inhibits the continuous advancement of pancreatic cancer cells in the G1, S, or G2 phases, inducing a condition of dormancy that prevents the malignant cells from obtaining the necessary resources for their growth (17). The ability to cause cell death through the production of ROS, cell proliferation, apoptosis, and cell cycle arrest, has generated significant interest among scientific community of SFN.

To explore the complex relationship between the basic mechanisms that cause pancreatic cancer, with a specific focus on the functions of reactive oxygen species (ROS), apoptosis, and cell cycle arrest have not been elucidated yet. SFN is a promising solution in the fight against pancreatic cancer, providing a versatile strategy to counter its continuous advancement (18). SFN exerts a fatal effect on pancreatic cancer cells by regulating cellular proliferation, inducing apoptosis, and regulating cell cycle arrest, ultimately leading to their mortality (19). As scientific study progresses, the complete therapeutic capabilities of SFN have the potential to greatly change the field of pancreatic cancer treatment, providing renewed optimism for both patients and medical professionals (18). The present study investigates the mechanism by which SFN induces apoptosis through the production of ROS and the modulation of several pathways. Nevertheless, it is necessary for us to further investigate the anticancer mechanism of SFN in relation to the activation of caspase-3 and the generation of ROS and γH2A.X in PC. Therefore, in the present study we elucidate the involvement of ROS in the anticancer effects generated by SFN, particularly in the process of apoptosis, in combination with the signaling of γH2A.X and caspase-3.

## 2 Materials and methods

### 2.1 Chemicals and reagents

Sulforaphane (SFN), DCF-DA, N-acetyl-L-cysteine (NAC), and propidium iodide (PI) were purchased from Sigma-Aldrich (St. Louis, MO, USA). JC-1 dye was from MedChemExpress (Monmouth Junction, NJ, USA). FBS and media were from BIOWEST (Nuaillé, France). EZ-CYTOX was from Daeil Lab Service (Seoul, Korea). Alexa Fluor 546, a goat anti-rabbit IgG secondary antibody was purchased from Thermo Fisher Scientific (Waltham, MS, USA).

### 2.2 Cell culture

MIA PaCa-2 and PANC-1 were purchased from Korean Cell Bank (KCLB, Seoul, Korea). Cells were cultured with DMEM high glucose containing 10% FBS, and 1% penicillin/streptomycin (Biowest, Nuaillé, France). Optimum culture condition was kept in cell incubator (37°C, humidified, 5% CO_2_).

### 2.3 Cytotoxicity assay

1.3x10^4^ of cells were seeded into 96-well plate and incubated at temperature 37°C and 5% for overnight in Co_2_ incubator. Substantially, each well was treated with various concentrations of SFN (12.5, 25, 50, 100 μM) for 24 h. Then, EZ-Cytox cell viability assay reagent (Daeil Lab Service, Seoul, Korea) was used according to the manufacturer’s manual. Microplate reader (Bio-Rad, Hercules, CA, USA) measured the absorbance at 450nm.

### 2.4 Colony formation assay

MIA PaCa-2 at cells density (1x10³/well) and PANC-1 (3x10^3^/well) were seeded onto 6-well plate and treated with 7.5 and 15 µM SFN for 24 h. After treatment, it was replaced with fresh media, and incubated for 9 days. The cells were washed two times with PBS, then fixed, and stained with Diff quick solution (Sysmex, Kobe, Japan).

### 2.5 Cell cycle analysis

Cells treated with SFN for 24 h were harvested with accutase^®^ (Innovative Cell Technologies, San Diego, CA, USA) and fixed with 70% cold ethanol for 2 h at 4°C. The cells were stained with propidium iodide (50 µg/mL) containing RNase A (10 µg/mL) for 15 min in the incubator. Cell cycle analysis (FL-2) was conducted with the FACS Calibur (Becton Dickinson, Bergen County, NJ, USA).

### 2.6 ROS assay

Cells treated with SFN for 24 h were stained with DCF-DA (20 µM) for 30min in the incubator. Then fluorescence analysis (FL-1) was conducted with the FACS Calibur (Becton Dickinson, Bergen County, NJ, USA).

### 2.7 Mitochondrial membrane potential analysis

After treatment with SFN for 24 h, cells were harvested with accutase^®^ (Innovative Cell Technologies, USA), and stained with JC-1 (2.5 µM) for 15 min in the incubator. JC-1 monomer (FL-1) and JC-1 aggregates (FL-2) were simultaneously measured with the FACS Calibur (Becton Dickinson, Bergen County, NJ, USA).

### 2.8 Caspase-3 activity assay

CaspGLOW™ Caspase-3 activity staining kit (BioVision, Milpitas, CA, USA) was used as manufacturer’s protocol. Briefly, cells were stained with FITC-DEVD-FMK for 30 min in the incubator. Cells resuspended in the enclosed buffer were analyzed (FL-1) by the FACSCalibur (Becton Dickinson, Bergen County, NJ, USA).

### 2.9 Immunofluorescence assay

MIA PaCa-2 and PANC-1 were seeded onto 4-well culture slide (SPL, Pocheon, Korea) and incubated for 24 h. After incubation, SFN was added into the slide glass for 24 h and washed with PBS. Cells were fixed and permeabilized with 3.5% paraformaldehyde and 0.1% Triton X-100, respectively. The slide glass was soaked with 2% BSA dissolved in PBS for 1 h, and then primary antibody (1: 1,000) overnight at 4 ° C. Secondary antibody corresponding primary antibody was labelled for 2 h. Mounting solution containing DAPI was used to mount, and each sample was visualized by the confocal microscopy FV10i (OLYMPUS, Tokyo, Japan).

### 2.10 Western blot analysis

MIA PaCa-2 and PANC-1 treated with SFN for 24 h were lysed with lysis buffer containing protease inhibitors (Translab, Daejeon, Korea) on the ice for 30 min. Cell lysate was centrifuged at 13,000 rpm for 10 min to collect supernatants. To load equal amount of protein, a BCA assay was performed. The proteins were loaded in each well in SDS-PAGE gel (6~15%) and separated under 100 V for 100 min, being transferred onto nitrocellulose membranes under 300 mA for 120 min. The Membranes were washed three times for 10 min with Tris-buffered saline containing 0.1% Tween 20 (TBST) and blocked with 5% skimmed milk dissolved in TBST for 1 h. The membranes were washed with TBST under same condition, then soaked in specific primary antibodies overnight; GAPDH (1: 1,000) (Santa Cruz Biotechnology, Dallas, TX, USA), γH2A.X (1: 1,000), cleaved caspase-3 (1: 1,000), cleaved PARP (1: 1,000, XIAP (1: 1,000) (Cell signaling, Beverly, MA, USA). The membranes were washed same as the former step and incubated with suitable secondary anti-mouse (Abcam, Cambridge, United Kingdom) or rabbit antibodies (Bioss Antibodies, Woburn, MA, USA) for 2 h at room temperature. Before protein identification, membranes were washed with TBST, and visualized by chemiluminescence imaging equipment (Davinch-K, Seoul, Korea).

### 2.11 Statistical analysis

The results are represented means ± standard deviation (SD). The statically significant values between control and treated group were attained by using Student’s t-test *, *p*<0.05; **, *p*<0.01; ***, *p*<0.001 versus untreated group.

## 3 Results

### 3.1 SFN exhibited cytotoxic effect on pancreatic cancer cell lines

**Figure 1:**
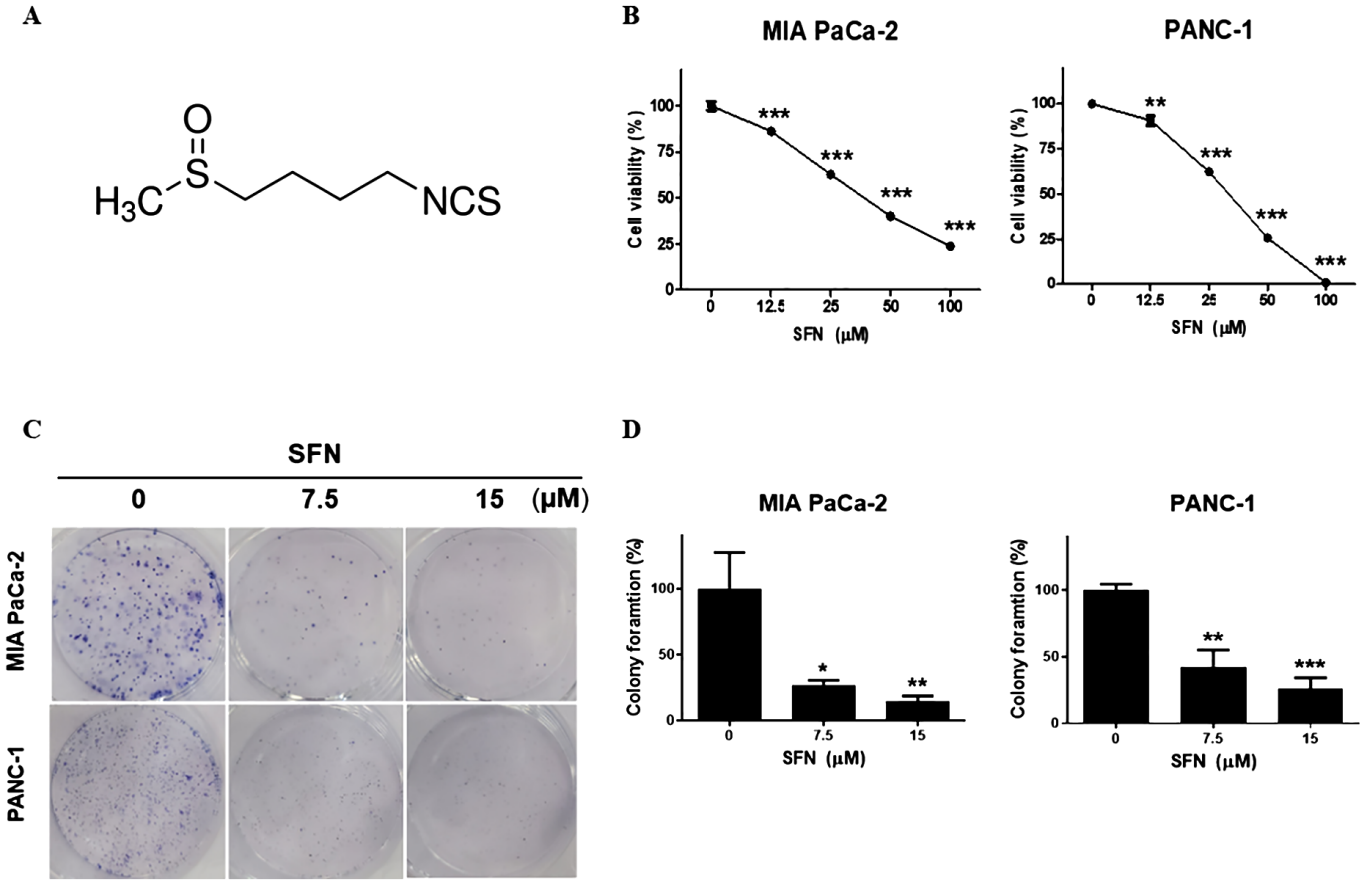
Cytotoxic effect of SFN on pancreatic cancer cell lines **(A)** Chemical structure of SFN **(B)** Cells were seeded onto 96-well plate and treated with indicated concentrations (0, 12.5, 25, 50 100 µM) of SFN for 24h. Cell viability was measured by EZ-Cytox, a cell viability test reagent. Cell viability data represent means ± SD. *p < 0.05, **p < 0.01, ***p < 0.001 versus previous concentration. **(C)** MIA PaCa-2 and PANC-1 cells were seeded onto 6-well plate, 1x10^3^ and 3x10^3^ respectively and treated with SFN (0, 7.5, 15 µM). SFN were replaced with fresh media and incubated for 9 days. Colonies were fixed and stained with Diff quick solution. **(D)** Bar graph represents the percentage of colony formation. Data represent the means ± SD. *p < 0.05, **p < 0.01, ***p < 0.001 versus untreated group.

Sulforaphane possesses various benefits for health, such as its ability to act as an antioxidant and reduce inflammation (9). Extensive research has been conducted on its capacity to inhibit cancer, protect against cardiovascular diseases, promote cognitive well-being, and facilitate detoxification mechanisms within the body (20). Sulforaphane (**Figure 1A**) has been shown to be an effective experimental therapy for pancreatic cancer. To evaluate the potential cytotoxic effects of SFN on pancreatic cancer cell lines, we exposed SFN to different concentrations (0, 12.5, 25, 50, 100 µM) and treated MIA PaCa-2 and PANC-1 cells. **Figure 1B** clearly shows that SFN caused a decrease in cell viability that was dependent on the dosage. As a result, more studies were conducted using doses of 0, 7.5, and 15 µM. **Figure 1C** demonstrates that SFN also impeded the ability to form colonies, confirming its inhibitory effect. Notably, there were noticeable distinctions between the control and SFN-treated groups, as depicted in **Figure 1D**. Therefore, SFN was found to be an inhibitory and cytotoxic activity in PC cells.

### 3.2 SFN increased the sub G_1_ ratio and induced apoptosis in pancreatic cancer cells

**Figure 2:**
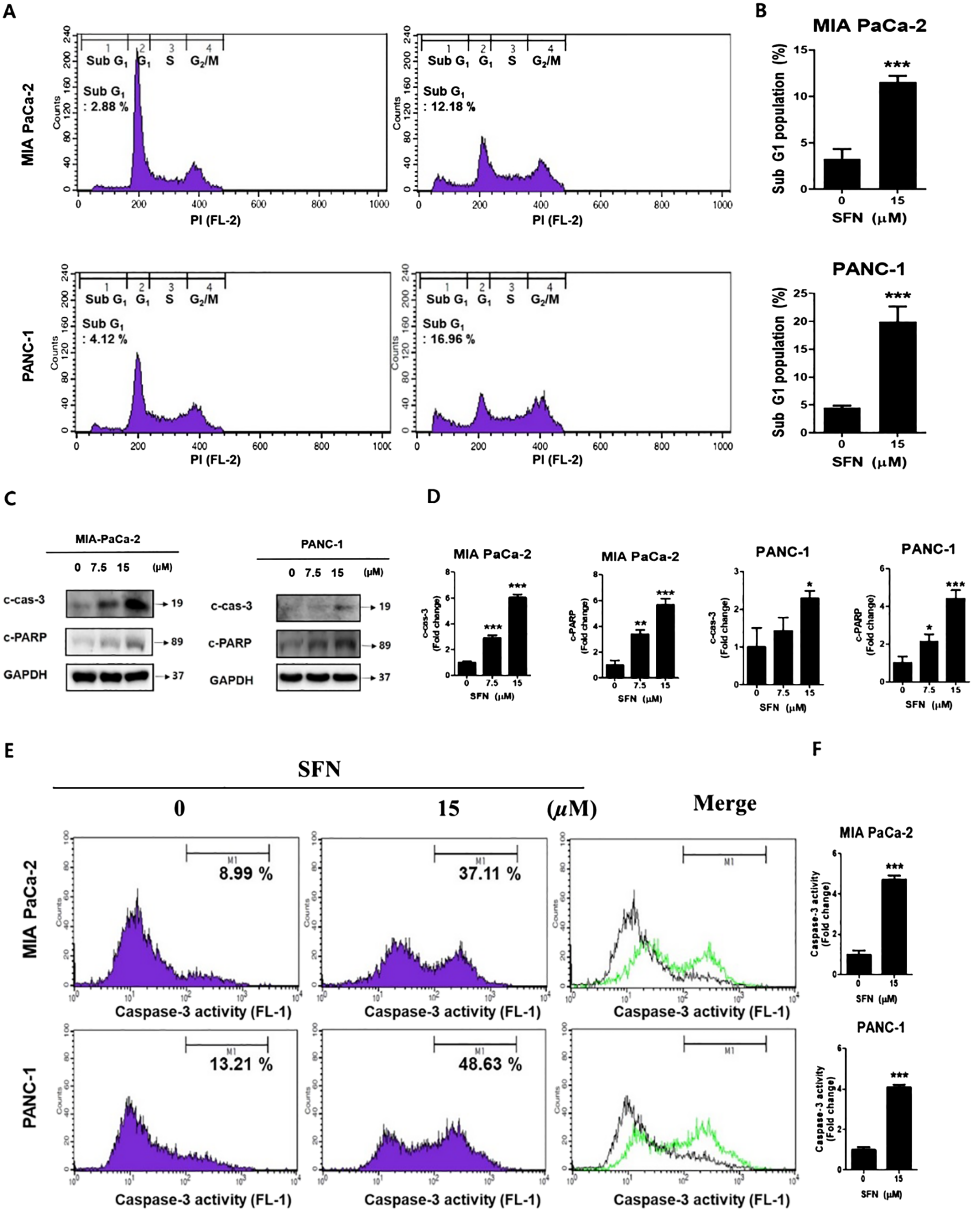
The apoptotic effect of SFN in pancreatic cancer cells **(A)** SFN-treated were fixed with 70% ethanol at 4 °C, stained with propidium iodide (PI), and analyzed (FL-2) by flow cytometry in MIA PaCa-2 and PANC-1 cells. **(B)** Bar graph represents the percentage of sub G_1_ population. **(C)** Protein extract from cell lysates was subjected to western blot for apoptotic markers cleaved caspase-3, and cleaved PARP in MIA PaCa-2 and PANC-1 cells. **(D)** Bar graph represents the ratio of protein. **(E)** MIA PaCa-2 and PANC-1 cells treated with SFN for 24 h were harvested, stained with FITC-DEVD-FMK, resuspended in the enclosed buffer, and analyzed (FL-1) by flow cytometry. **(F)** Bar graph represents the ratio of caspase-3 activity. Data represent the means ± SD. *p < 0.05, **p < 0.01, ***p < 0.001 versus untreated group.

A DNA intercalator called propidium iodide (PI) was utilized to conduct an analysis of the ratio of the sub G1 stage, which is indicative of cell death. In both pancreatic cancer cells, SFN was shown to have a considerable rise in the sub G1 population (**Figures 2A, B**). To determine whether the rise in the sub G1 population is indicative of apoptosis, a Western blot performed the analysis. Consequently, there was an increase in the number of cleaved forms of caspase-3 and PARP (**Figures 2C, D**). Additionally, it was demonstrated that the activity of caspa-se-3 was seen to be greatly elevated (**Figures 2E, F**). Therefore, these results suggest that SFN induces a G1 cell cycle arrest and elevates the level of γH2AX protein, which is indicative of DNA damage.

### 3.3 SFN caused changes in mitochondrial membrane potential

**Figure 3:**
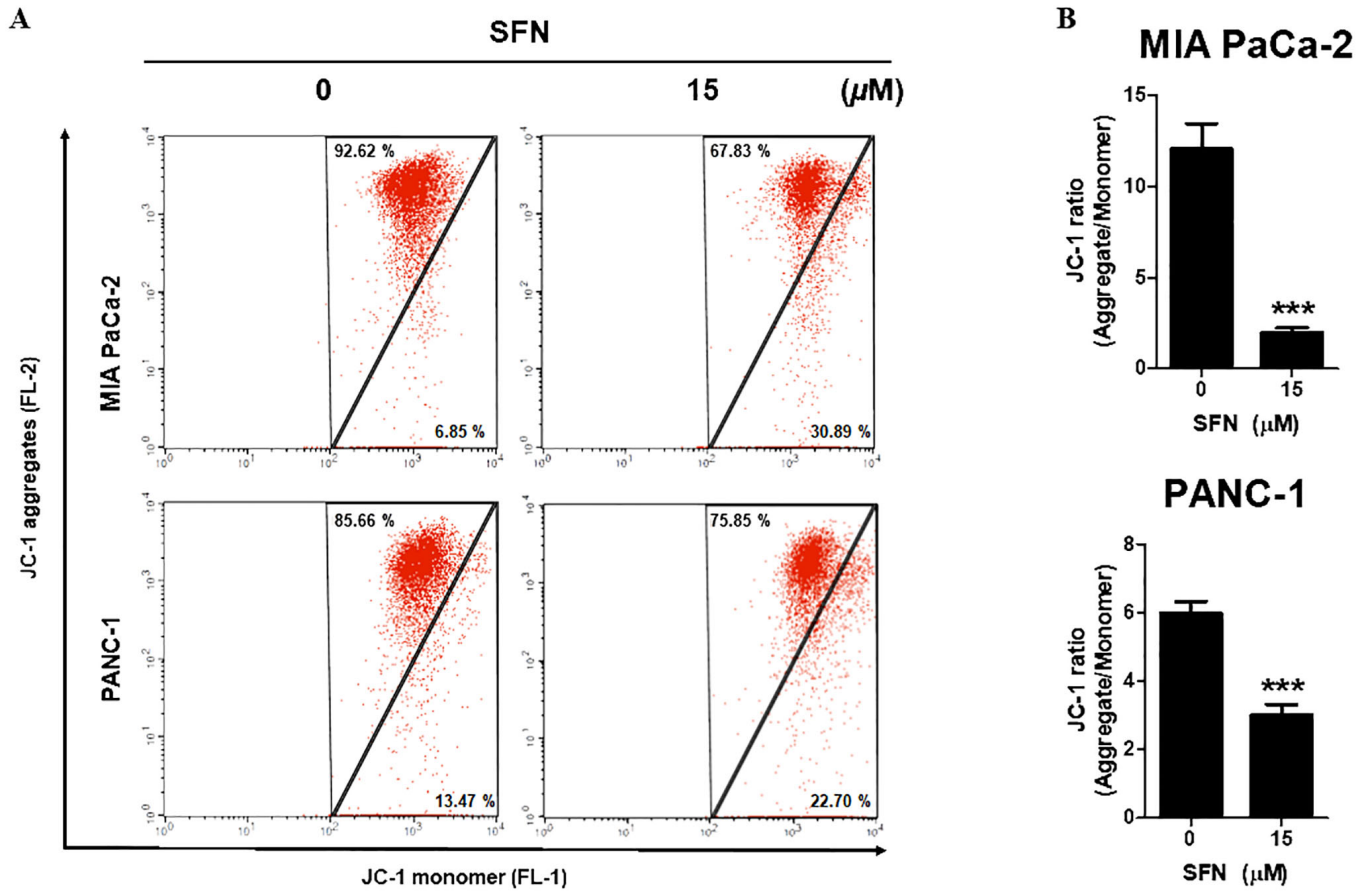
Effect of SFN on mitochondrial membrane potential (ΔΨm) in pancreatic cancer cells. **(A)** MIA PaCa-2 and PANC-1 cells were treated with SFN for 24 h, stained with JC-1 and analyzed (FL-1 and FL-2) by flow cytometry. **(B)** Bar graph represents the ratio of JC-1 ratio (Aggregates/Monomer) in both pancreatic cancer cells. Data represents means ± SD. ***p < 0.001 versus untreated group.

Since mitochondria are crucial for the survival of cells, the decrease of mitochondrial membrane potential (ΔΨm) is regarded as an indication of apoptosis (21). To determine the change in ΔΨm, the staining process was carried out using JC-1, a cationic dye that displays either a green (unhealthy) or red (healthy) coloration depending on the ΔΨm. As can be shown in **Figure 3A**, SFN is responsible for a reduction in JC-1 aggregates (red, healthy) while simultaneously increasing JC-1 monomer (green, unhealthy). SFN was responsible for the reduction in the red-to-green ratio of JC-1, as shown in **Figure 3B**. Therefore, these results indicate that SFN induced alterations in the mitochondrial membrane potential in PC.

### 3.4 SFN caused DNA damage with up-regulated γH2A.X in pancreatic cancer cells

**Figure 4:**
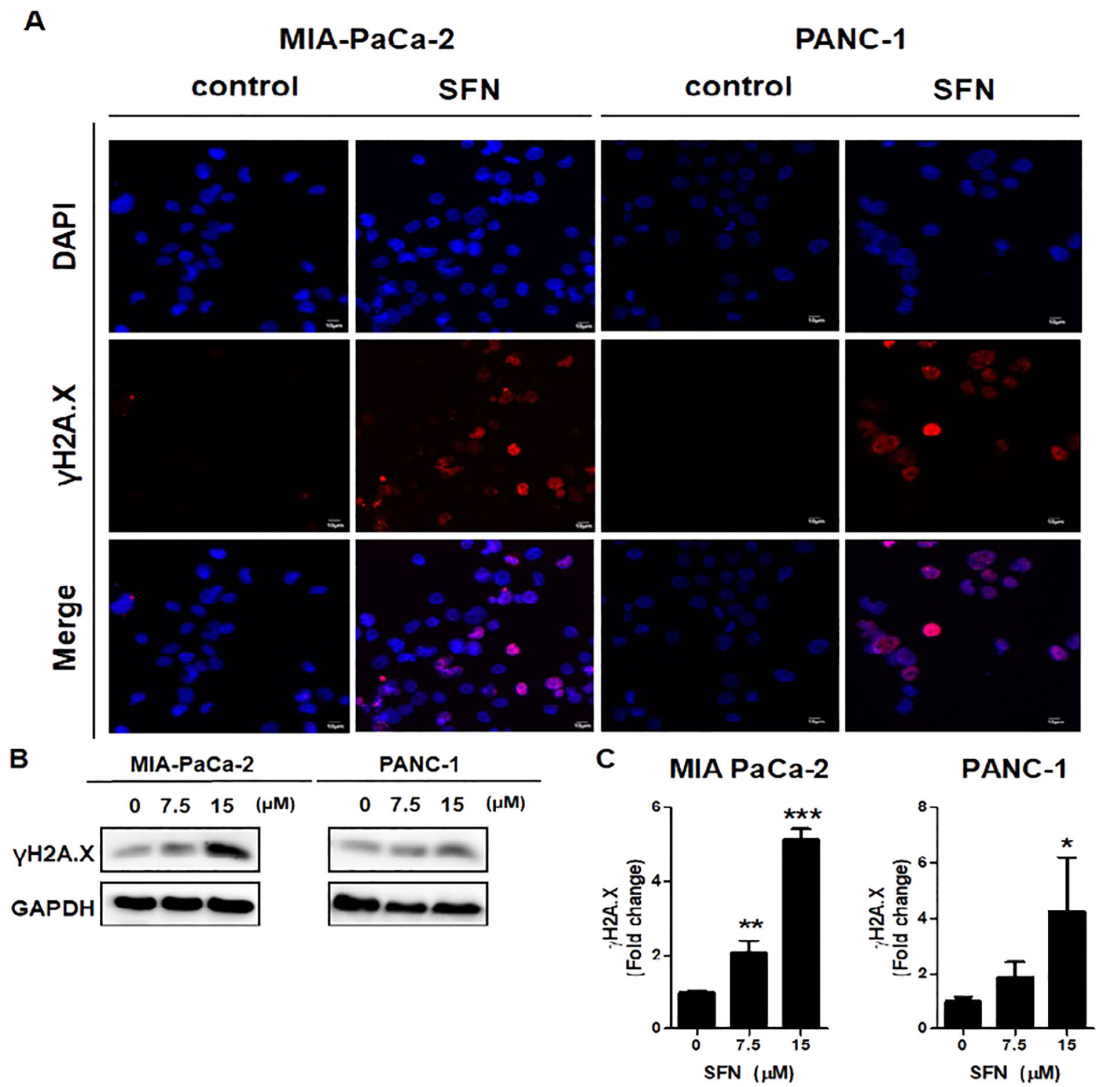
Induction of DNA damage by SFN) in pancreatic cancer cells. **(A)** MIA PaCa-2 and PANC-1 cells were incubated with primary antibody, followed by Alexa Fluor 546-conjugated-secondary antibody (Red) for immunofluorescence assay. The samples were mounted with mounting solution containing DAPI (Blue). Images were taken by the confocal microscopy FV10i (OLYMPUS, Tokyo, Japan). **(B)** Protein extract from cell lysates was subjected to western blot for γH2A.X in MIA PaCa-2 and PANC-1 cells. **(C)** Bar graph represents the ratio of γH2A.X expression in both pancreatic cancer cells. Data represents means ± SD. *p < 0.05, **p < 0.01, ***p < 0.001 versus untreated group.

DNA damage was recognized to have a positive correlation with the process of apoptosis (22). Here, we examine that SFN has been demonstrated to induce DNA damage in pancreatic cancer cells, as indicated by the increased expression of γH2A.X, a biomarker for DNA double-strand breaks. Therefore, to determine if SFN causes DNA damage in MIA PaCa-2 and PANC-1 cells, an immuno-fluorescence test (IF) and a western blot for γH2A.X, which is a marker for DNA damage, were carried out. The results that were displayed in IF images demonstrated that SFN is responsible for inducing DNA damage while simultaneously elevating γH2A.X levels in MIA PaCa-2 and PANC-1 cells (**Figure 4A**). Western blot data, in conjunction with pictures obtained from immunofluorescence (IF), demonstrated an increase in the level of γH2A.X expression in both PaCa-2 and PANC-1 cells from MIA-Paca-2 (**Figures 4B, C**). Therefore, this data implied that SFN induced DNA damage in pancreatic cancer cells, resulting in increased levels of γH2A.X.

### 3.5 Reactive oxygen species play pivotal role in SFN-induced apoptosis in pancreatic cancer cells

**Figure 5:**
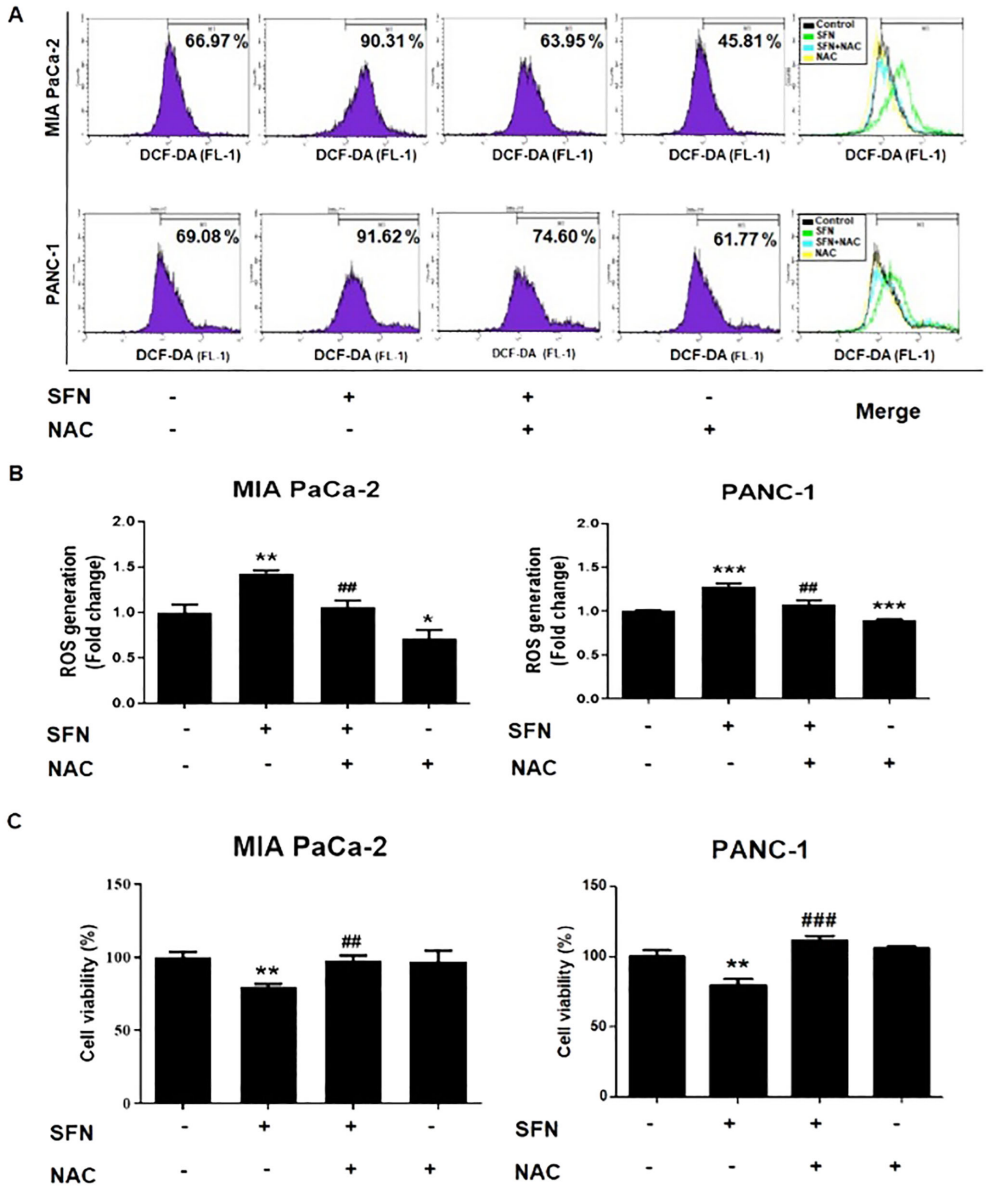
ROS generation from SFN and its relationship with cell viability in pancreatic cancer cells. **(A)** MIA PaCa-2 and PANC-1 cells were seeded onto 6-well plate and then subjected to the indicated treatments. The pH change from 1mM NAC was adjusted by adding sodium hydroxide for cell culture. Cells were stained with DCF-DA and flow cytometry (FL-1) was performed to evaluate samples. **(B)** Bar graph represents the ratio of ROS generation. **(C)** MIA PaCa-2 and PANC-1 cells were seeded onto 96-well plate and subjected to cell viability assay. The pH change from 1mM NAC was adjusted by adding sodium hydroxide for cell culture. Cell viability was measured by EZ-Cytox, a cell viability test reagent. Data represents means ± SD. *p < 0.05, **p < 0.01, ***p < 0.001 versus untreated group. #*p* < 0.05, ##*p* < 0.01, ###*p* < 0.001 versus SFN only treated group.

ROS is widely acknowledged to have a significant role in the modulation of biological processes (23). Staining with 2’,7’-dichlorofluorescin diacetate (DCF-DA) was utilized to assess the formation of reactive ROS, which are also accountable for the process of apoptosis (24). Therefore, SFN caused a considerable increase in ROS in MIA PaCa-2 and PANC-1 cells as compared to the control group (**Figure 5A**). In addition, N-acetyl-L-cysteine (NAC), which is often employed as a ROS scavenger, was co-treated to verify whether SFN causes the production of ROS and, consequently, apoptosis. Because of this, NAC was able to reduce the production of ROS and the cytotoxic effect in MIA PaCa-2 and PANC-1 cells (**Figures 5A-C**). Thus, ROS are crucial in the process of SFN-induced apoptosis in pancreatic cancer cells.

### 3.6 NAC reversed SFN-induced changes of X-linked inhibitor of apoptosis protein and γH2A.X in pancreatic cancer cells

**Figure 6:**
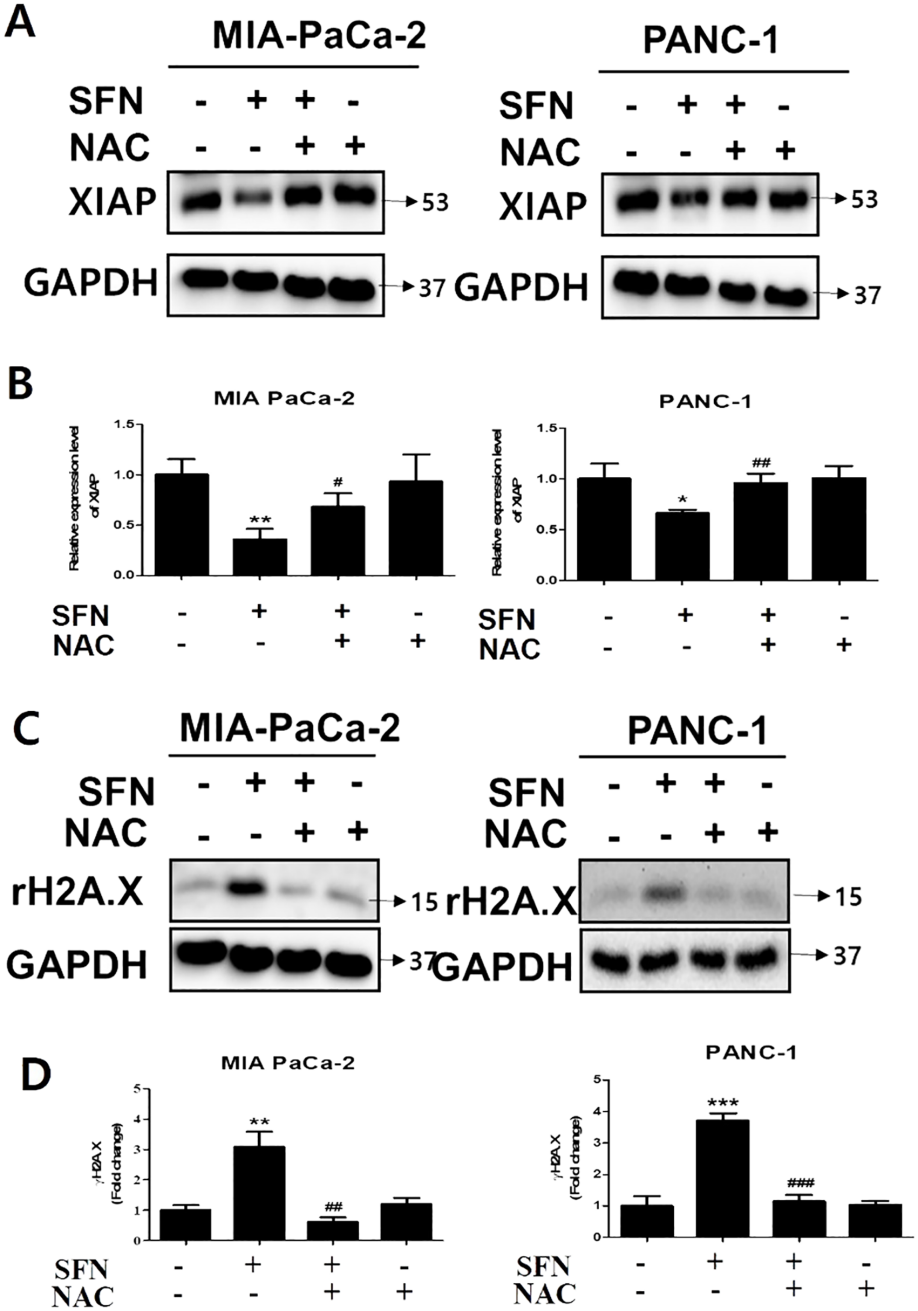
The changes in SFN-induced XIAP and γH2A.X by NAC in pancreatic cancer cells. **(A)** Protein extract from cell lysates was subjected to western blot for XIAP in MIA PaCa-2 and PANC-1 cells. **(B)** Bar graph represents the ratio of XIAP expression in both pancreatic cancer cells. **(C)** Protein extract from cell lysates was subjected to western blot for γH2A.X in MIA PaCa-2 and PANC-1 cells. **(D)** Bar graph represents the ratio of γH2A.X expression. Data represents means ± SD. *p < 0.05, **p < 0.01, ***p < 0.001 versus untreated group. #*p* < 0.05, ##*p* < 0.01, ###*p* < 0.001 versus SFN only treated group.

By binding to substrate through the baculoviral IAP repeat (BIR) domain, the X-linked inhibitor of apoptosis protein (XIAP), which is a member of the inhibitor of apoptosis proteins (IAPs), can suppress the action of caspase-3 (25). Because caspase-3 is an essential component in the execution of apoptosis, the development of a complex between XIAP and caspase-3 prevents proteins that are necessary for apoptosis from being accessed (26). SFN was shown to diminish the expression of XIAP, which was then reversed by NAC, which is a ROS scavenger, in both pancreatic cancer cells (**Figures 6A, B**). In addition, the effect of NAC on the change in the amount of γH2A.X expression in MIA PaCa-2 and PANC-1 cells was reduced, as shown in **Figures 6C, D**. Therefore, our results indicate that NAC counteracted the alterations generated by SFN in X-linked inhibitor of apoptosis protein and γH2A.X levels in pancreatic cancer cells.

## 4 Discussion

Pancreatic cancer constitutes one of the deadliest types of cancer, with a high fatality rate and few treatments available. As a result, there is an urgent need to investigate novel medicines that might effectively target pancreatic cancer cells. Sulforaphane, a naturally occurring chemical found in cruciferous vegetables, has emerged as a potential option for anticancer therapy (27). In this present study, we would like to observe at recent research on the mechanism by which sulforaphane causes cell death in pancreatic cancer cells, with a focus on the role of ROS and the effects on cell proliferation, DNA damage, mitochondrial function, cell cycle arrest, and apoptosis.

Perspectives from pharmacological as well as toxicological experimental investigations on sulforaphane, a compound that has the potential to be beneficial to health in terms of preventing and treating diseases (28). SFN was found to have a cytotoxic effect on pancreatic cancer cell lines (29). The significance of this discovery lies in the fact that it sheds light on the potential of SFN as a therapeutic agent for the treatment of pancreatic cancer. Chen et al. (2018) found that HUVECs, used as normal control cells, did not show significant inhibitory effects at SFN concentrations below 20 μM (30). Thus, 7.5 and 15 μM SFN were selected for further experiments in our study. Additionally, studies on human primary salivary fibroblasts, human gingival epithelial progenitor cells, and a human salivary gland acinar cell line (NS-SV-AC) demonstrated no morphological changes at SFN concentrations below 14 μM (31). There was no significant difference between combined treatments and standalone effects of CIS or 5-FU on these cells. This indicates that normal mesenchymal and epithelial cells are not adversely affected by SFN, while the viability of immortalized or malignant cells is reduced. In the current study, we found that administration of SFN resulted in a dose-dependent reduction in cell viability and hindered the formation of colonies, so validating its inhibitory impact in PC (**Figure 1**). An established connection between apoptosis as well as the cell cycle can be made by observing the striking resemblance in physical characteristics between mitosis and apoptosis (32). Flow cytometric DNA analysis of PC cells revealed the existence of sub G1 populations, indicating the presence of cells undergoing apoptosis, as well as the inhibition of cell cycle progression in the S-phase and G2/M-phase (33). It have been found that both SFN and RT exhibit a significant and dose-dependent decrease in cell survival and induce a G2/M cell cycle arrest and raise the level of γH2AX protein, which is an indicator of DNA damage (34). However, in the present investigation SFN has demonstrated the ability to enhance the sub G1 ratio, which is a reliable indicator of apoptosis in pancreatic cancer cells (**Figure 2**). Additionally, caspase-3 is cleaved at the Asp175 location, a process that is aided by granzyme B or caspase-10. The cleavage process gives rise to the p20 and p11 subunits, which then triggers the activation of caspase-3 (35). Upon activation, caspase-3 specifically identifies and breaks down a range of intracellular proteins that contribute to the structure and function of the cell, including poly (ADP-ribose) polymerase (PARP), gelsolin, and DNA-dependent kinase. This process ultimately results in the death of the cell (36). In our study we found that assessing apoptosis through caspase-3 activity and the expression of apoptosis markers like cleaved caspase-3 and PARP, SFN unequivocally triggered apoptosis. (**Figure 2**). This evaluation aimed to determine if the cell cycle arrest induced by SFN was associated with apoptosis. Therefore, our findings indicate that sulforaphane triggers cell death by activating apoptotic mechanisms.

Mitochondria have a crucial function in controlling apoptosis, and changes in mitochondrial membrane potential can initiate the process of apoptosis (37, 38). Oxygen serves as the ultimate receiver of electrons in the process of energy production within the complex respiratory chain of mitochondria (39). Regulation of mitochondrial transition pores facilitates the movement of proteins from mitochondria to the cytoplasm, thereby controlling apoptosis (40, 41). Past studies have indicated that a decrease in mitochondrial membrane potential (ΔΨm) is a sign of potential apoptosis in cells (21). Consistently, we found that SFN altered the ratio of JC-1 (a fluorescent dye) monomers to aggregates, indicating a significant reduction in ΔΨm. Thus, SFN’s anticancer effects might stem from inducing mitochondrial dysfunction and altering membrane integrity in MIA PaCa-2 and PANC-1 cells (**Figure 3**). The study demonstrates that sulforaphane induces alterations in the mitochondrial membrane potential of pancreatic cancer cells, providing additional evidence for its ability to trigger apoptosis.

Cancer is characterized by DNA damage, therefore causing DNA damage in cancer cells might result in their death (42). The histone H2A.X plays a crucial role in managing DNA damage (43). H2A.X phosphorylation triggers many DNA repair mechanisms and has important functions in cellular control (44). The phosphorylation status of H2A.X is a crucial indicator of genome integrity and provides insights into DNA-related processes in cells and tissues (45). The phosphorylation status of H2A.X serves as a reliable biomarker for DNA damage, genotoxicity, and clinical indicators such as radiation outcome, medication effectiveness, and tissue regeneration (46). It is a valuable biomarker for several present and future biomedical uses. Here, we examined that western blotting and immunofluorescence analyses demonstrated that SFN treatment induced DNA damage in MIA Pa-Ca-2 and PANC-1 cells, highlighting the crucial role of the γH2A.X signaling pathway in SFN’s anticancer efficacy (**Figure 4**). This demonstrates an additional method via which sulforaphane exerts its harmful effects on cells.

Reactive oxygen species (ROS), which include molecules like superoxide, hydrogen peroxide, and hydroxyl radicals, have been identified as regulators of different cellular processes, including apoptosis (47). Nevertheless, the role of ROS in pancreatic cancer is both beneficial and detrimental (48). ROS-induced DNA damage facilitates the onset of carcinogenesis and the conversion of cells into a malignant state (49). Simultaneously, reactive oxygen species (ROS) can act as signaling molecules, promoting both cell survival and the advancement of cancer (50). Conversely, an excessive amount of reactive oxygen species (ROS) causes the release of cytochrome c into the cytoplasm, which then initiates apoptosis (51–53). In the current study, we demonstrate that SFN triggers the production of ROS, which causes oxidative stress, resulting in harm to cells and ultimately leading to cell death (**Figure 5**). Similarly, SFN markedly elevated ROS production and reduced cell viability, a phenomenon mitigated by N-acetyl-L-cysteine (NAC). This implies that ROS serves as a pivotal factor in the SFN-triggered apoptosis in MIA PaCa-2 and PANC-1 cells (**Figure 5**). Therefore, this study suggests that ROS play a crucial role in the process of SFN-induced apoptosis in pancreatic cancer cells.

The X-linked inhibitor of apoptosis protein (XIAP) regulates apoptosis via interacting with caspase-3 (54). XIAP contains the baculoviral inhibitor of apoptosis repeat (BIR) domain, which allows it to bind with caspase and block the activation of caspase-3, which is responsible for carrying out apoptosis (55). Directly inhibiting XIAP activity shows potential as a therapeutic strategy for pancreatic cancer, namely by targeting the XIAP/caspase axis (56). Additionally, N-acetylcysteine (NAC) is a powerful antioxidant that may effectively remove reactive oxygen species (ROS) and alleviate oxidative stress (57). In our study shows that NAC can counteract the effects of sulforaphane on the levels of X-linked inhibitor of apoptosis protein and γH2A.X in pancreatic cancer cells. This provides additional evidence for the involvement of ROS in mediating the harmful effects of SFN and emphasizes the potential usefulness of antioxidant treatments in reducing its effects. Moreover, in MIA PaCa-2 and PANC-1 cells, SFN markedly reduced XIAP, exhibiting an anti-apoptotic trait, while concurrently boosting the expression of apoptotic proteins like γH2A.X in a dose-dependent manner (**Figure 6**). This effect was reversed by NAC, suggesting a potential pathway for SFN-induced apoptosis after DNA damage (**Figure 6**). SFN induces apoptosis triggered by double-strand breaks (DSBs), occurring notably during the subG1 phase of the cell cycle, indicating its apoptotic nature. It’s imperative to recognize that SFN’s efficacy in pancreatic cancer isn’t uniform, despite its extensive use in research involving MIA PaCa-2 and PANC-1 cells, due to the complex regulatory pathways within the human body. In essence, the intricacies of these regulatory pathways *in vivo* surpass those observed *in vitro*. Therefore, these findings underscore the impact of SFN on the cytotoxicity and apoptosis of pancreatic cancer cells.

Lastly and eventually, SFN, a bioactive compound found in cruciferous vegetables such as broccoli, has been shown to have relatively low oral bioavailability (58). This is primarily due to its rapid metabolism and excretion. After oral ingestion, SFN is absorbed in the gastrointestinal tract, but its bioavailability is limited by its metabolism to less active forms and its quick clearance from the body (59). Recent studies indicate that SFN is metabolized in the liver and excreted primarily through urine, which can impact its systemic availability and biological effects (60). Strategies to enhance its bioavailability, such as using formulations that improve its absorption or combining it with other compounds, are currently being investigated to maximize its therapeutic potential.

## 5 Conclusion

**Figure 7:**
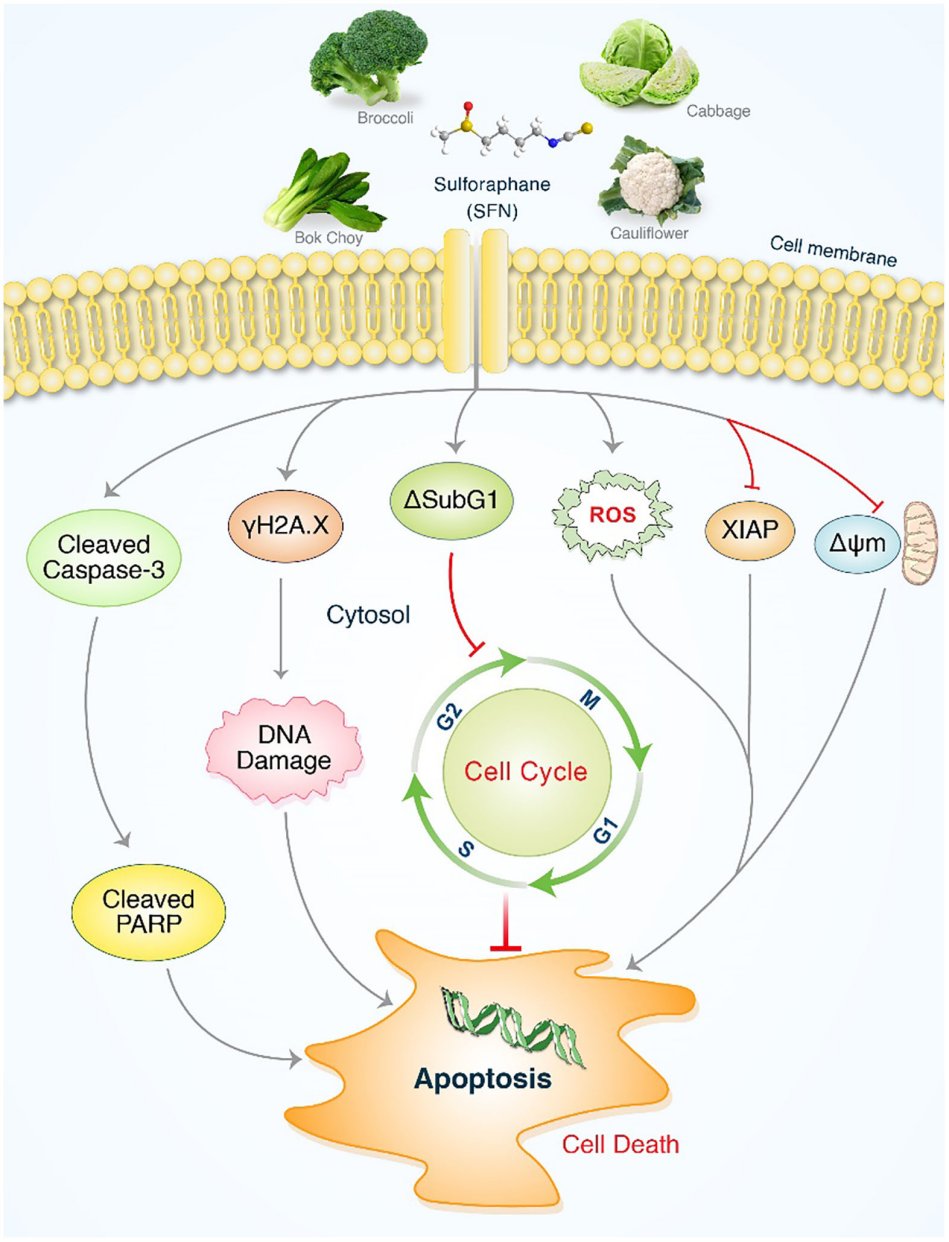
An illustrative depiction of the impact of SFN on pancreatic cancer cell lines. Evidence of SFN-induced stimulation of apoptosis in pancreatic cancer cells. SFN significantly enhanced the production of reactive oxygen species (ROS), leading to the activation of cleaved PARP and cleaved caspases-3, as well as sub G1 accumulation. This ultimately resulted in apoptosis through cell cycle arrest and the activation of γH2A.X. After undergoing SFN treatment, the levels of XIAP and ΔΨm were reduced in pancreatic cancer cells, resulting in the initiation of apoptosis.

It has been demonstrated that SFN has a considerable impact on MIA PaCa-2 and PANC-1 cells. Significantly, SFN therapy greatly increased programmed cell death mediated by ROS in proteins such as cleaved PARP. This also resulted in the activation of caspases, leading to the production of cleaved caspase-3 and the suppression of XIAP, which in turn triggered H2A.X. SFN enhanced DNA damage and cytotoxicity against MIA PaCa-2 and PANC-1 cells through the ROS mediated pathway, as depicted in **Figure 7**. Moreover, the involvement of antioxidant systems and ROS signaling is crucial for the advancement of pancreatic cancer and its responsiveness to cancer therapies. Additionally, ROS signaling plays a crucial role in the management of pancreatic cancer and its response to cancer treatments. SFN could serve as a new target in future strategies and medications for controlling levels of ROS in t of pancreatic cancer.

## Data Availability

The original contributions presented in the study are included in the article/supplementary material. Further inquiries can be directed to the corresponding author.
